# Multicenter Retrospective Analysis of Turkish Patients with Chronic Myeloproliferative Neoplasms

**DOI:** 10.4274/tjh.2016.0005

**Published:** 2017-03-01

**Authors:** Nur Soyer, İbrahim C. Haznedaroğlu, Melda Cömert, Demet Çekdemir, Mehmet Yılmaz, Ali Ünal, Gülsüm Çağlıyan, Oktay Bilgir, Osman İlhan, Füsun Özdemirkıran, Emin Kaya, Fahri Şahin, Filiz Vural, Güray Saydam

**Affiliations:** 1 Ege University Faculty of Medicine, Department of Hematology, İzmir, Turkey; 2 Hacettepe University Faculty of Medicine, Department of Hematology, Ankara, Turkey; 3 Sakarya University Training and Research Hospital, Clinic of Hematology, Sakarya, Turkey; 4 Gaziantep University Faculty of Medicine, Department of Hematology, Gaziantep, Turkey; 5 Erciyes University Faculty of Medicine, Department of Hematology, Kayseri, Turkey; 6 İzmir Bozyaka Training and Research Hospital, Clinic of Hematology, İzmir, Turkey; 7 Ankara University Faculty of Medicine Hospital, Department of Hematology, Ankara, Turkey; 8 İzmir Atatürk Training and Research Hospital, Clinic of Hematology, İzmir, Turkey; 9 İnönü University Faculty of Medicine Hospital, Department of Hematology, Malatya, Turkey

**Keywords:** Chronic myeloproliferative neoplasms, treatment, Survival, JAK2 mutation

## Abstract

**Objective:**

Chronic myeloproliferative neoplasms (CMPNs) that include polycythemia vera (PV), essential thrombocythemia (ET), and primary myelofibrosis (PMF) are Philadelphia-negative malignancies characterized by a clonal proliferation of one or several lineages. The aim of this report was to determine the demographic features, disease characteristics, treatment strategies, and survival rates of patients with CMPNs in Turkey.

**Materials and Methods:**

Across all of Turkey, 9 centers were enrolled in the study. We retrospectively evaluated 708 CMPN patients’ results including 390 with ET, 213 with PV, and 105 with PMF.

**Results:**

The JAK2V617F mutation was found positive in 86% of patients with PV, in 51.5% of patients with ET, and in 50.4% of patients with PMF. Thrombosis and bleeding at diagnosis occurred in 20.6% and 7.5% of PV patients, 15.1% and 9% of ET patients, and 9.5% and 10.4% of PMF patients, respectively. Six hundred and eight patients (85.9%) received cytoreductive therapy. The most commonly used drug was hydroxyurea (89.6%). Leukemic and fibrotic transformations occurred at rates of 0.6% and 13.2%. The estimated overall survival in PV, ET, and PMF patients was 89.7%, 85%, and 82.5% at 10 years, respectively. There were no significant differences between survival in ET, PV, and PMF patients at 10 years.

**Conclusion:**

Our patients’ results are generally compatible with the literature findings, except for the relatively high survival rate in PMF patients. Hydroxyurea was the most commonly used cytoreductive therapy. Our study reflects the demographic features, patient characteristics, treatments, and survival rates of Turkish CMPN patients.

## Abstract (tr)

**Amaç:**

Polisitemia vera (PV), esansiyel trombositemi (ET) ve primer miyelofibrozu (PMF) içeren kronik miyeloproliferatif neoplaziler (KMPN), bir ya da birden fazla serinin klonal proliferasyonu ile karakterize Philadelphia kromozomu negatif olan malignitelerdir. Bu çalışmanın amacı, Türkiye’de KMPN’li hastaların demografik özellikleri, hastalık karakteristikleri, tedavi stratejileri ve yaşam oranlarını belirlemektir.

**Gereç ve Yöntemler:**

Türkiye’nin her yanından 9 merkez çalışmaya katıldı. Biz geriye dönük olarak ET’li 390, PV’li 213 ve PMF’li 105 hasta olmak üzere toplam 708 KMPN’li hastanın verisini değerlendirdik.

**Bulgular:**

JAK-2 mutasyonu PV’li hastaların %86’sında, ET’li hastaların %51,5’inde ve PMF’li hastaların %50,4’ünde pozitif bulundu. Tanıda tromboz ve kanama, PV’li hastaların sırasıyla %20,6 ve %7,5’inde, ET’li hastaların %15,1 ve %9’unda ve PMF’li hastaların %9,5 ve %10,4’ünde saptandı. Altı yüz sekiz hasta (%85,9) sitoredüktif tedavi almıştı. En sık kullanılan ilaç hidroksiüre (%89,6) idi. Lösemik ve fibrotik transformasyon sıklığı %0,6 ve %13,2 idi. 10 yıllık hesaplanan toplam sağkalım PV, ET ve PMF hastalarında sırasıyla %89,7, %85 ve %82,5 idi. 10 yıllık toplam sağkalım açısından ET, PV ve PMF hastalarında anlamlı fark yoktu.

**Sonuç:**

Sonuçlarımız, PMF hastalarının yüksek sağkalımı hariç literatürle benzerdir. Hidroksiüre ülkemizdeki en sık kullanılan sitoredüktif ajandır. Bizim çalışmamız, Türk KMPN hastalarının demografik özelliklerini, hastaların karakteristiklerini, tedavilerini ve sağkalım oranlarını yansıtmaktadır.

## INTRODUCTION

Chronic myeloproliferative neoplasms (CMPNs) are Philadelphia-negative malignancies characterized by a clonal proliferation of one or several lineages. According to the World Health Organization (WHO) classification, CMPNs include polycythemia vera (PV), essential thrombocythemia (ET), and primary myelofibrosis (PMF) [1]. Their natural history is marked by thrombohemorrhagic complications and a propensity to transform into myelofibrosis and acute leukemia [2].

The JAK2V617F mutation is present in a majority of PV patients (90%-98%), whereas only about 50% of patients with ET and PMF are affected [3,4]. Mutations other than JAK2 are calreticulin (CALR) and the myeloproliferative leukemia (MPL) virus oncogene. CALR mutations occur in 25%-35% of patients with PMF and 15%-24% with ET. These are rarely seen in PV. MPL mutations occur in 4% of ET patients, 8% of PMF patients, and rarely in PV [5].

Thrombotic complications have been reported in 30%-50% of PV cases and 11%-45% of ET cases [6,7,8]. The incidence of cardiovascular complications was found to be higher in PV patients aged >65 years or with a history of thrombosis than in younger subjects with no history of thrombosis [9]. Leukocytosis was found to be an independent risk factor for arterial thrombosis in both PV and ET [10,11,12,13]. Thrombotic complications have been reported in between 7.2% and 11% of PMF patients [14,15]. Bleeding complications are less common than thrombotic complications in PV. They were reported in 4.2% of 1545 patients with PV and in 3%-25.7% of patients with ET [16,17].

Long-term survival in CMPNs is significantly shorter compared to control populations. In a large study, median survivals were approximately 20 years for ET, 14 years for PV, and 6 years for PMF. The incidence of leukemic transformation was 3.8% for ET, 6.8% for PV, and 14.2% for PMF. Fibrotic transformation rates were reported as 10.3% in ET and 12.5% in PV [18]. History of thrombosis, leukocytosis, and advanced age are responsible for poor survival in both PV and ET [19,20,21,22]. In PMF, poor survival is predicted by advanced age, leukocytosis, anemia, transfusion dependency, thrombocytopenia, circulating blasts, constitutional symptoms, and unfavorable karyotypes [23].

Current treatment in ET and PV is directed primarily at minimizing the risk of thrombosis and secondarily at alleviating vasomotor symptoms. According to these goals, patients with PV and ET are stratified into risk categories and the treatment is tailored to the patient’s risk group [5]. Low-dose aspirin, hydroxyurea, interferon-α, and anagrelide can be used for the treatment of PV and ET. In PMF, the International Prognostic Scoring System (IPSS) and Dynamic IPSS are used for assessing survival at diagnosis and at any time in the disease course, respectively. Therapy is planned according to patients’ risk groups [4].

There has not been a large multicenter study that evaluated the demographic features, treatments, and survival of patients with CMPNs in Turkey. The aim of this report was to determine the demographic features, patient characteristics, treatments, and survival rates of patients with CMPNs in Turkey.

## MATERIALS AND METHODS

This study was designed as a retrospective multicenter study from Turkey and was approved by the Ege University Ethics Committee (Number 13-5.1/6). Across all of Turkey, 9 centers were enrolled in the study. The primary objective of the study was to evaluate the demographic features, treatments, and survival of patients with CMPNs in Turkey. For data collection from the centers, a case report form was prepared by the primary investigator. This form consisted of demographic features and patient characteristics, laboratory data at diagnosis, treatments, and the last status of patients. The case report forms were completed by each center’s investigators.

Patients of ≥18 years old with the diagnosis of PV, ET, or PMF according to WHO criteria were enrolled in the study [24]. Each center reevaluated their patients who were diagnosed before acceptance of the WHO criteria. The study population was also selected based on the availability of clinical and laboratory information at the time of initial diagnosis. Patients were excluded if they did not fulfill WHO criteria for PV, ET, or PMF and if they did not attend follow-ups regularly.

Major arterial thrombosis included transient ischemic attacks, thrombotic cerebrovascular accidents, angina pectoris, myocardial infarction, and peripheral arterial thromboembolism. Major venous thrombosis included deep venous thrombosis of the peripheral vasculature, pulmonary embolism, and abdominal vein thrombosis. Bleeding events included gastrointestinal tract bleeding, intracerebral hemorrhage, and soft tissue hematoma. Cardiovascular risk factors included hypertension, tobacco use, diabetes mellitus, and hyperlipidemia.

Patients who were diagnosed with PV without a JAK2V617F assay were evaluated as “not available” (NA) patients. If we excluded NA patients from the analysis, JAK2 mutation status was evaluated in only verified PV patients that had a JAK2V617F assay.

Risk factors of PMF patients were evaluated with the IPSS at diagnosis [25]. ET and PV patients were classified into high-risk and low-risk categories according to their age and history of thrombosis [26].

Treatment data were obtained according to specific therapies including cytoreductive therapy, antiplatelet therapy, androgens, steroids, thalidomide, erythropoiesis-stimulating agents, splenectomy, ruxolitinib, and red blood cell transfusions. If there was more than one specific treatment in the patient’s history, these therapies were also recorded. If allogeneic stem cell transplantation was performed it was also recorded.

Leukemic transformation was defined according to the WHO criteria for acute leukemia [24]. The WHO diagnostic criteria for PMF were applied to assign the disease transformation into post-PV and post-ET myeloproliferative categories.

### Statistical Analysis

All the statistical analyses were performed by using the data obtained from the patients’ files. Demographic and disease characteristics of the patients were summarized for all patients using descriptive statistics.

Statistical analyses were performed using SPSS 16.0 and Excel 2007. The variables were first assessed by Kolmogorov-Smirnov/Shapiro-Wilk testing in terms of normal distribution. The results were provided as mean ± standard deviation for normally distributed variables and as median (minimum-maximum) for abnormally distributed parameters. All analyses were based on the laboratory parameters obtained at the time of diagnosis. All p-values were two-tailed and statistical significance was set at the level of p<0.05.

Overall survival (OS) was defined as the time period between the time of diagnosis and death because of any reason or last contact. OS evaluation was performed by using the Kaplan-Meier method.

## RESULTS

Seven hundred and eight patients who were diagnosed between 1987 and 2014 were included in the study; 55.1% of all patients had ET, 30.1% had PV, and 14.8% had PMF. The JAK2V617F mutation was found positive in 75.1% of patients with PV, in 51.5% of patients with ET, and in 50.4% of patients with PMF. After exclusion of NA patients with PV, the JAK2V617F mutation was found in 86%. MPL mutation was observed in only 3 (2.6%) of 115 patients with ET. We did not detect MPL mutation in other groups.

At diagnosis, thrombosis was observed in 20.65% of PV, 15.12% of ET, and 9.5% of PMF patients and bleeding occurred in 7.5% of PV, 9% of ET, and 10.4% of PMF patients. Thrombosis and bleeding at diagnosis were observed in 21.9% and 7.5% of verified PV patients, respectively. Secondary malignancy history was obtained from 10 (1.44%) of 691 patients at diagnosis. Patients’ clinical and hematological data at diagnosis are shown in Table 1. Cardiovascular risk factors were determined in 258 (42.8%) of 603 patients with ET and PV.

Six hundred and eight patients (85.9%) had been treated with cytoreductive therapy. The most commonly used drug was hydroxyurea (89.6%). Antiplatelet therapy was used in 553 (78.1%) patients. Treatment choices and risk stratification of patients are shown in Table 2. In PV patients, 13.2% of 213 were treated with only therapeutic phlebotomy and antiplatelet therapy. In ET patients, 7.5% of 390 were treated with only antiplatelet therapy and 1.8% of 390 patients were observed without any treatment. In PMF patients, 34.3% of 105 did not receive cytoreductive treatment. Ten (27.8%) of 36 received antiplatelet therapy, 15 (41.7%) of them received red blood cell transfusions, and the others (30.5%) were observed without any treatment. Cytoreductive therapy was changed in 195 patients for various reasons. In second-line treatment, hydroxyurea was changed to anagrelide in 147 patients. Anagrelide and interferon were changed to hydroxyurea in 5 and 9 patients, respectively. Hydroxyurea and anagrelide were changed to interferon in 30 patients and 1 patient, respectively. Three patients were treated with ruxolitinib after hydroxyurea treatment. Two patients and 9 patients received androgen therapy and steroid therapy, respectively. Erythropoiesis-stimulating agents were administered to 3 patients. Red blood cell transfusion was performed in 79 patients. Splenectomy was performed in 10 (1.4%) patients. One patient was treated with splenic radiotherapy. Twelve PMF patients (11.4%) were treated with allogeneic stem cell transplantation.

During follow-up, secondary malignancy was determined in 7 (0.9%) patients [3 ET (0.7%) and 4 PV (1.9%) patients]. Three and 2 of 7 patients were receiving hydroxyurea and anagrelide, respectively. Other patients did not use any cytoreductive therapy. Leukemic transformation was observed in 4 (0.6%) of all patients. Progression to myelofibrosis was observed in 80 (13.2%) of 603 PV and ET patients. At the end of the study and data collection period, 35 patients were deceased, 648 patients were still alive, and 25 patients had interruptions in their follow-up.

**Figure 1:**
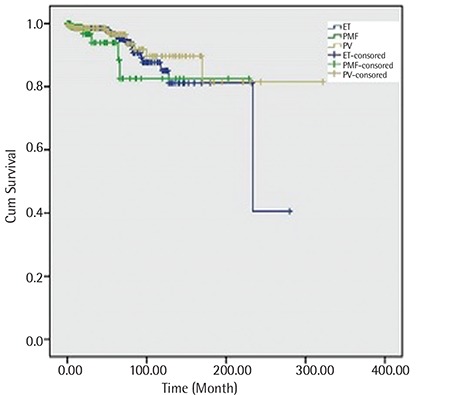
Overall survival of chronic myeloproliferative neoplasm patients. ET: Essential thrombocythemia, PMF: primary myelofibrosis, PV: polycythemia vera.

**Figure 2:**
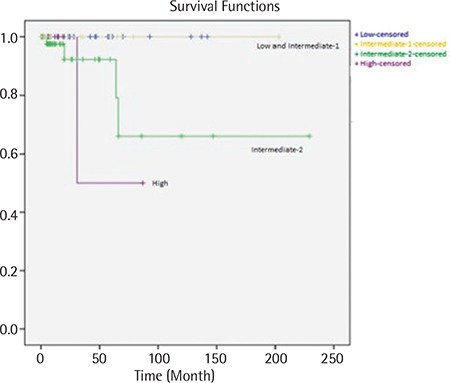
Overall survival of primary myelofibrosis patients according to the International Prognostic Scoring System.

The median follow-up was 38 months (range: 0-322) and the estimated OS was 86.7% at 10 years in all patients. Among the 213 PV patients, the median follow-up was 49 months (range: 0-322) and the estimated OS was 89.7% at 10 years. In the verified PV group, the estimated OS was 89.4% at 10 years. In 390 ET patients, the median follow-up was 39 months (range: 0-280) and the estimated OS was 85% at 10 years. Among the 105 PMF patients, the median follow-up was 19 months (range: 0-229) and the estimated OS was 82.5% at 10 years (Figure 1). According to the IPSS, the estimated OS was 100% in the low and intermediate-1 risk group, 92.4% in the intermediate-2 risk group, and 50% in the high risk group at 5 years in PMF (Figure 2). There were no significant differences between survival rates in ET, PV, and PMF patients at 10 years.

## DISCUSSION

The aim of this study was to determine the demographic features, patient characteristics, treatments, and survival rates of patients with CMPNs in Turkey. We evaluated 708 patients from 9 centers across all of Turkey. This study was planned as a multicenter and retrospective trial so that we might evaluate CMPN practices in Turkey.

In our study, the incidence of JAK2 mutation, the history of thrombosis, and the median age at diagnosis were lower than in the literature [3,4,9]. After excluding NA patients from analysis, the incidence of JAK2 mutation (86%) was closer to that of other studies. The incidence of bleeding was comparable to that reported in the literature [6,16]. Thrombotic complications were reported in 30% to 50% of PV patients in other studies [6,7]. Epidemiological studies have reported that the incidence of thrombosis increases with age [27]. Lower median age at diagnosis might be related to a lower incidence of thrombosis. Almost all patients with PV harbor a JAK2 mutation that includes JAK2V617F and JAK2 exon 12 [4]. Additionally, JAK2 exon 12 mutation has been associated with younger age at diagnosis [28]. The lower incidence of JAK2V617F in our series might be associated with younger age at diagnosis, preanalytical mistakes, and problems of laboratory analysis, such as the exon 12 mutation not being analyzed in all centers routinely. The type of sample, the cellular fraction of the sample, and the nucleic acid template were considered for the detection of the JAK2 mutation. Additionally, some qualitative methods like the Sanger sequencing method used for detection of the JAK2 mutation underestimated the number of patients harboring the mutation [29]. The inability of qualitative assays to identify those patients with lower allele burdens is another problem. All of these factors seem to be related to low JAK2 mutation positivity.

Another issue is that we determined a low hemoglobin value at diagnosis in one patient who was diagnosed with PV. This patient was evaluated because of portal vein thrombosis and at the time of evaluation had gastrointestinal bleeding due to warfarin use. JAK2V617F mutation was found positive.

MPL mutation was reported in approximately 4% of ET patients and 8% of PMF patients [5]. The frequency of MPL mutation in ET patients (2.6%) was similar to rates reported in the literature. We did not detect MPL mutations in our PMF and PV patients. These results might be related to the low number of patients who were detected with this mutation.

In our ET patients, median age at diagnosis, the incidence of thrombosis and bleeding at diagnosis, and JAK2 mutation positivity were compatible with literature findings [4,8,17]. In our PMF patients, the incidence of thrombosis and bleeding at diagnosis and JAK2 mutation positivity were compatible with the literature but median age at diagnosis was slightly higher [14,15,18].

The estimated OS was 86.7% at 10 years in our CMPN patients. The 10-year and 3-year OS of CMPN patients was 72% and 80%, respectively [30,31]. The 10-year OS in PV patients was reported to be between 56% and 83% [32,33]. In our ET and PV patients, OS rates were similar to those of previous studies. The 10-year OS in PMF patients (21%-46%) was significantly worse than that of patients with ET or PV [32,33]. In our PMF patients, we found that the 10-year survival was 82.5% with a median 19 months of follow-up. It is important that 71.4% of PMF patients in our study had low or intermediate-1 risk. This finding might explain our high survival rate in PMF patients.

Previous studies suggested that survival in myeloproliferative neoplasm patients can be influenced by several factors such as increased age, male sex, and PMF subtype of CMPN, which are associated with decreased survival in myeloproliferative neoplasms [30,31,32,34,35]. Geography and ethnicity can also impact survival [32,36].

Although approximately 50% of patients with ET and PV were classified into the low risk group according to risk stratification in our series, 86.8% of PV patients and 90.7% of ET patients received cytoreductive therapy. This might be associated with cardiovascular risk factors that were determined in 42.8% of 603 patients with ET and PV.

The incidence of secondary malignancy was reported to be between 8% and 20% [37,38]. Our secondary malignancy incidence was lower than that in the literature. It is possible that secondary malignancies were underestimated in our series.

In our study, the leukemic transformation rate was lower than in the literature. Leukemic and fibrotic transformation rates were reported as 3.8%-14.2% in CMPNs and 10.3%-12.5% in ET and PV, respectively [18]. The low leukemic transformation rate might be associated with several factors. First, this was a retrospective study, so some data were not found because of inadequate records. Second, PMF incidence is highest in elderly patients. In our study, patients were excluded if they did not have regular follow-ups and this resulted in the exclusion of some patients diagnosed with CMPNs at older ages who could not attend regular appointments because of socioeconomic conditions.

## CONCLUSION

Our patients’ results are generally compatible with literature findings, except for the relatively high survival rate in PMF patients. Hydroxyurea was the most commonly used cytoreductive therapy in our study. This study reflects the demographic features, patient characteristics, treatments, and survival rates of patients with CMPNs in Turkey.

## Figures and Tables

**Table 1:**
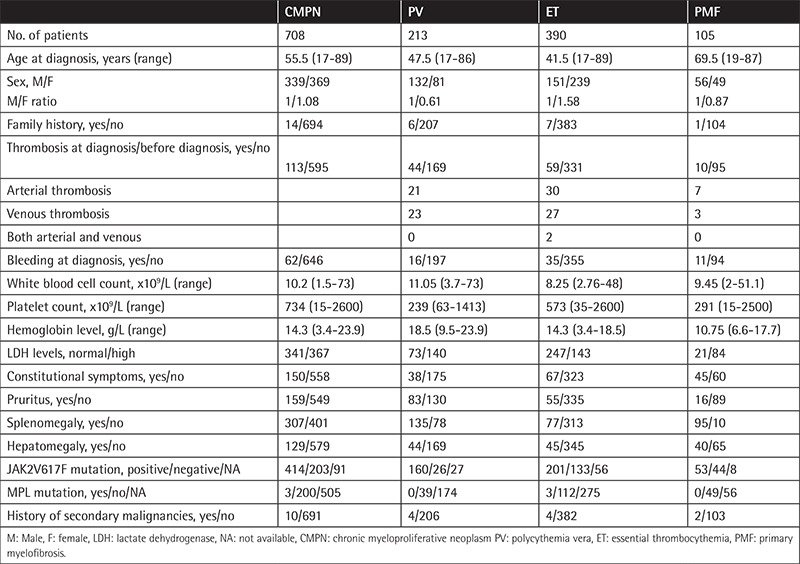
Clinical and hematological data of patients at diagnosis.

**Table 2:**
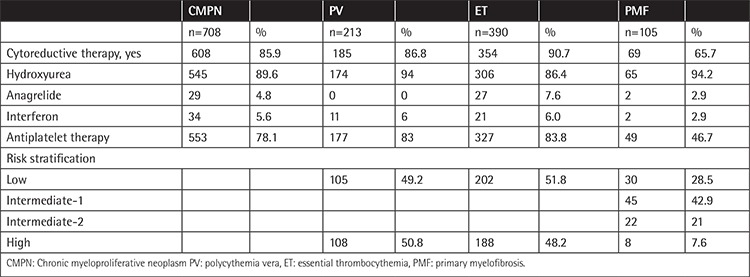
First-line treatment choices and risk stratification of patients.
